# Functional connectivity between the thalamus and the primary somatosensory cortex in major depressive disorder: a resting-state fMRI study

**DOI:** 10.1186/s12888-018-1913-6

**Published:** 2018-10-19

**Authors:** Lijun Kang, Aixia Zhang, Ning Sun, Penghong Liu, Chunxia Yang, Gaizhi Li, Zhifen Liu, Yanfang Wang, Kerang Zhang

**Affiliations:** 1 Department of Psychiatry, First Hospital of Shanxi Medical University, Taiyuan, 030001 People’s Republic of China; 2 Shanxi Medical University, Taiyuan, 030001 People’s Republic of China

**Keywords:** Major depressive disorder, Functional connectivity, Thalamus, Primary somatosensory cortex, SI-thalamic functional connectivity

## Abstract

**Background:**

Studies have confirmed that the thalamus and the primary somatosensory cortex (SI) are associated with cognitive function. These two brain regions are closely related in structure and function. The interactions between SI and the thalamus are of crucial significance for the cognitive process. Patients with major depressive disorder (MDD) have significant cognitive impairment. Based on these observations, we used resting-state functional magnetic resonance imaging (rs-fMRI) to investigate whether there is an abnormality in the SI-thalamic functional connection in MDD. Furthermore, we explored the clinical symptoms related to this abnormality.

**Methods:**

We included 31 patients with first-episode major depressive disorder and 28 age-, gender-, and education-matched healthy controls (HC). The SI-thalamic functional connectivity was compared between the MDD and HC groups. The correlation analyses were performed between areas with abnormal connectivity and clinical characteristics.

**Results:**

Compared with healthy subjects, the MDD patients had enhanced functional connectivity between the thalamus and SI (*p* < 0.05, corrected). Brain areas with significantly different levels of connectivity had a negative correlation with the Assessment of Neuropsychological Status total score (*r* = − 0.383, *p* = 0.033), delayed memory score (*r* = − 0.376, *p* = 0.037) and two-digit continuous operation test score (*r* = − 0.369, *p* = 0.041) in MDD patients.

**Conclusions:**

These results demonstrate that SI-thalamic functional connectivity is abnormal and associated with the core clinical symptoms in MDD. The SI-thalamic functional connectivity functions as a neurobiological feature and potential biomarker for MDD.

## Background

Major depressive disorder (MDD) is a prevalent psychiatric disorder [1]. MDD is characterized by affective disorder, vegetative symptoms, and cognitive function deficits. Clinicians and researchers have found that MDD patients are characterized by significantly poorer cognitive functions, including attention, executive functioning, episodic memory, and processing speed [2–4]. The evidence suggests that cognitive dysfunction is observed beyond the acute phase, and some of these impairments even persist during illness remission, which may lead to depressive relapse and dysfunction [5–7]. Researchers have proposed that functional and mood symptom recovery must include cognitive function recovery [8].

The thalamus is the hub of cortical-subcortical connections located deep in the brain and has been traditionally viewed as a simple relay station for neurons for all body sensations (except olfaction) to then project to the cerebral cortex [9]. The thalamus works as a tightly connected “central core” of brain regions for multiple tasks and behaviours [10]. Some evidence suggests that the thalamus combined with the dorsal anterior cingulate cortex and anterior insular compose the salience network, which has been described as having a central role in cognitive control [11, 12]. Recent research has shown that the thalamus can amplify and sustain cortical representations, and a new framework acknowledging thalamus-frontal circuits in cognition has been defined [13]. In disease conditions, increased thalamic functional connectivity was related to decreased cognitive function [14–18]. These findings suggest that the importance of the thalamus in cognitive activity is gradually being recognized.

The primary somatosensory cortex (SI) is located in the postcentral gyrus and structurally and functionally connected to the thalamus [19, 20]. SI is viewed as an acquisition and transformation sensory signal structure; meanwhile, involvement in controlling and modulating associatively learned behaviors is becoming apparent [21–24]. SI participated in the non-painful stimulation encoding process, and furthermore, SI maintained task-related tactile information in the late maintenance stage and contributed to the memory trace of a pain sensation [25–28]. A researcher even suggested SI as an “embodied mind” that makes the unconscious self exist in concert with the embodied facet of the self [29].

All of this evidence provides promising opportunities for understanding the importance of the thalamus and SI in cognitive function. These two brain regions are closely related in structure and function. The interactions between SI and the thalamus are of crucial significance for the cognitive process. In MDD, patients have cognitive impairments. Therefore, we hypothesized that abnormal SI-thalamic functional connectivity might be a neurobiological feature of MDD that is closely related to these clinical symptoms. Resting-state functional magnetic resonance imaging (rs-fMRI) is a powerful neuroimaging technique that enables researchers to measure spontaneous fluctuations in activity among distinct brain regions [30]. The method of using rs-fMRI to explore the brain’s intrinsic functional networks has been called resting-state functional connectivity (rs-FC) and has been used in numerous studies [31, 32]. Therefore, we used rs-FC as our experimental measure. This study may generate a new understanding of the underlying neurobiology depressive disorders.

## Methods

### Participants

Thirty-one patients with first-episode major depressive disorder were recruited from the First Hospital of Shanxi Medical University. At least two consultant psychiatrists diagnosed all patients according to the criteria for MDD in the “Diagnostic and Statistical Manual of Mental Disorders Fourth Edition (DSM-IV)” and the Chinese version of the Modified Structured Clinical Interview for DSM-IV TR Axis I Disorders Patient Edition (SCID-I/P). The patients were excluded if they met any of the following criteria: 1) had comorbid mental or neurological illnesses or personality disorders; 2) had used psychiatric drugs in the previous 2 weeks; 3) scored less than 17 on the 17-item Hamilton depressive scale (HAMD-17) administered by well-trained research assistants with backgrounds in psychology or psychiatry; 4) were pregnant; 5) were under 18 or over 60 years of age; 6) had a history of substance abuse or drug addiction; or 7) were left-handed or mixed-handed. We collected the patients’ general information, including name, gender, age, education level, occupation and family history of psychotic diseases.

Twenty-eight age-, gender-, and education-matched healthy controls were recruited for comparison. Participants were excluded if they met any of the following criteria: 1) had mental or neurological illnesses or personality disorders; 2) had a history of substance abuse or drug addiction; 3) had a family history of mental disorders; 4) were pregnant; 5) were under 18 or over 60 years of age; or 6) were left-handed or mixed-handed.

The Ethical Committee for Medicine of the First Hospital of Shanxi Medical University approved this study. Written informed consent was received from all participants prior to inclusion.

### Clinical assessment

An increasing number of studies have shown that depression cannot be observed from the single point of view of depression but must be observed from the multiple perspectives of affective and somatization symptoms and cognition [33]. We used the HAMD-17 and the Snaith-Hamilton Pleasure Scale (SHAPS) to assess affective experience. We used the fatigue severity scale (FSS) to assess somatization symptoms. Finally, we used the Repeatable Battery for the Assessment of Neuropsychological Status (RBANS) and Continuous Performance Test (CPT) to assess cognition features. Our senior psychologist evaluated all the scales in this study.

The HAMD-17 is the most commonly used scale for assessing the severity of MDD, and there is a good degree of confidence in the scale’s validity and reliability [34].

The SHAPS is a 14-item scale, each item ranged from 0 to 3 (“strongly agree” to “disagree”), and it was filled out by the subject to evaluate the clinical absence of pleasure [35].

The FSS is a 9-item scale of fatigue severity, each item ranged from the lightest (i.e., 1) to the worst (i.e., 7), and evaluated clinically elevated fatigue [36].

The RBANS is a neuropsychological screening scale designed by Randolph in 1998 for ease of use and for rating neuropsychological function in 20 ~ 89-year-old people and has excellent reliability and validity [37]. The scale has 12 test items covering the five factors of immediate memory, visuospatial/constructional, language, attention, and delayed memory.

The CPT mainly examines the continuous attention level of the participants, the constant concentration on the response, and the level of arousal. For this task, different numbers are presented on the display screen in random order, asking the participants to identify when the same number appeared repeatedly and to react within a specified time. Based on the number of digits of the flashing figures, it is divided into two-digit, three-digit, and four-digit categories [38].

### MRI acquisition and preprocessing of rs-fMRI data

#### MRI acquisition

A MAGNETOM Trio Tim 3.0 T scanner (Siemens Medical Solutions, Germany) with a 12-channel birdcage head coil located at the Shanxi Provincial People’s Hospital was used to acquire rs-fMRI. rs-fMRI was performed using an echo planar imaging (EPI) sequence with the following parameters: TR = 2000 ms, TE = 30 ms, flip angle = 70°, FOV = 24 × 24 cm, matrix = 64 × 64, slice gap = 2 mm, slice thickness = 2 mm, 6 min acquisition. During the resting functional scan, participants were instructed to keep their eyes closed and let their minds wander and not to fall asleep; all participants reported that they did not fall asleep.

#### Preprocessing of rs-fMRI data

Data preprocessing was conducted using the DPARSFA toolbox version 3.2, which was based on statistical parametric mapping 8 (SPM8) and the Resting-State fMRI Data Analysis Toolkit (REST) [39, 40]. The first 10 volumes of functional time points were discarded to allow the participants to adapt to the scanning noise. The remaining 170 volumes were preprocessed, which included the following: 1) slice timing, 2) realigning to reduce head motion (all head movements exceeding 2 mm were excluded), 3) spatial normalizing to the Montreal Neurological Institute (MNI) coordinate space with 3 × 3 × 3 mm, 4) spatial smoothing with a 6 × 6 × 6 full-width at half maximum (FWHM) kernel, 5) linear detrending, 6) temporal bandpass filtering (0.01–0.08 Hz), and 7) white matter signal, cerebrospinal fluid signal, global mean signal and six head motion parameters were used as covariates.

Regions of interest (ROI) were obtained with the WFU Pick Atlas 3.0.5, which automatically generates segmented atlas ROI templates in MNI space [41, 42]. The thalamus was defined as both “Thalamus_L” and “Thalamus_R”. SI was defined as “Brodmann areas 3, 1, 2”. Then, the image was resliced to the dimension of our functional images (voxel dimension: 3x3x3).

Connectivity between the thalamus and SI was examined using DPARSFA. The mean time course for the thalamus ROI was calculated by averaging the time course for all voxels within the thalamus ROI [43]. Calculations of the functional connection of the thalamus to the whole brain were made. The resulting correlation coefficients were transformed into z scores by Fisher’s z transform to create subject-specific maps [44]. Then, we performed a two-sample t-test across the two groups of subjects for the primary somatosensory cortex ROI.

### Data analysis

All analyses were performed using IBM SPSS Statistics Version 23.0 (SPSS 23.0). Data are expressed as the means ± standard deviations (SD). Independent-sample t tests and *χ*^*2*^ tests were used to analyse the demographic data of the two groups. A two-sample t-test was conducted to compare the clinical symptoms scores across groups. *P*-values of 0.05 were considered statistically significant (two-tailed).

The rs-FC of the HC and MDD groups was contrasted using the DPARSFA toolbox version 3.2. The two-sample t-test was used to identify abnormalities between the two groups. To ensure that effects were not accounted for by other factors, such as age, sex and years of education, these variables were included as regressors of no interest. Multiple comparison corrections were determined by Monte Carlo simulation (1000 iterations) using the REST AlphaSim program [45]. Voxels with *p* < 0.01 and cluster size ≥4 voxels (108 mm^3^) were regarded as brain areas with a significant difference which confined within the SI mask, corresponding to corrected *p* < 0.05 [46]. From the preprocessed resting-state fMRI data, we extracted the mean time course of each of the statistically significant clusters.

Furthermore, we used Pearson’s correlation coefficients to evaluate any relationships between abnormal clusters and clinical features of MDD patients by SPSS 23.0. The results were considered statistically significant at *p* < 0.05.

## Results

### Demographic data

As shown in Table 1, the mean age (mean ± SD) of the patients and the control group were 29.96 ± 9.73 and 26.79 ± 6.91 years, respectively. No differences in age, sex or years of education were observed between the patients and controls (*p* < 0.05). Table 1 also shows the HAMD-17, SHAPS, FSS, RBANS, and CPT scores for each group. All of these differences were significant between the patients and controls.

**Table 1:** Demographic and Clinical characteristics of participants

Characteristics (mean ± SD)	MDD patients(*n* = 31)	Healthy controls(*n* = 28)	*p*
Gender (male/female)	21/10	17/11	0.581
Age(years)	29.96 ± 9.73	26.79 ± 6.91	0.157
Educated(years)	13.74 ± 2.43	14.86 ± 2.43	0.084
HRSD17 score	20.26 ± 3.19	4.00 ± 1.09	< 0.001^*^
SHAPS total score	23.06 ± 5.97	4.59 ± 4.29	< 0.001^*^
FSS total score	45.83 ± 12.54	23.93 ± 5.58	< 0.001^*^
RBANS			
immediate memory	73.34 ± 15.43	95.48 ± 14.98	< 0.001^*^
visuospatial/constructional	88.78 ± 19.49	102.00 ± 14.71	0.009^*^
language	86.26 ± 17.06	97.52 ± 15.72	.0100^*^
attention	98.61 ± 15.79	119.56 ± 13.06	< 0.001^*^
delayed memory	83.13 ± 14.10	93.56 ± 10.68	0.013^*^
subpoint	82.30 ± 15.69	101.67 ± 11.99	< 0.001^*^
CPT			
two digits	2.38 ± 0.93	3.15 ± 0.82	0.002^*^
three digits	1.57 ± 0.94	2.74 ± 0.91	< 0.001^*^
four digits	0.94 ± 0.60	1.70 ± 0.76	< 0.001^*^
average	1.77 ± 1.08	2.53 ± 0.70	0.007^*^

*p < 0.05

### Functional connectivity analysis results

**Fig. 1:**
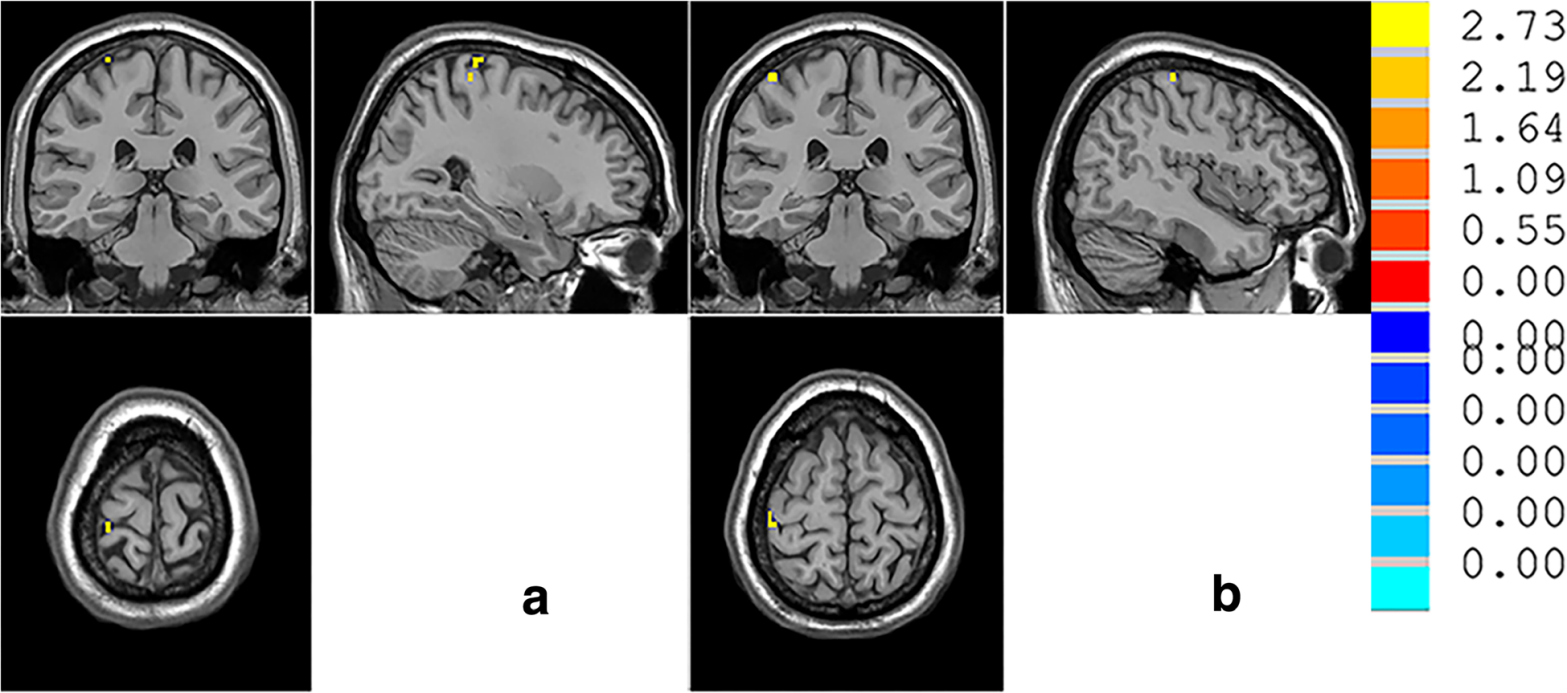
The images display the regions in the right central posterior gyrus that show increased functional connectivity from the thalamus in major depressive disorder (MDD) compared to healthy controls (HC) at rest. The colour bar represents the range of T values

Compared with the HC group, the MDD group showed significant decreases in rs-FC between the thalamus and SI in two clusters. As shown in Fig. 1 and Table 2, both clusters were in the right central posterior gyrus: the first cluster had the maximally intense voxel at MNI coordinates x = 27, y = − 33, z = 75 (8 voxels, about 216 mm^3^, *T* = 3.6162, *p* < 0.05 AlphaSim correction), and the second cluster had the maximally intense voxel at MNI coordinates x = 45, y = − 30, z = 63 (8 voxels, about 216 mm^3^, *T* = 3.2803, *p* < 0.05 AlphaSim correction).

**Table 2:** Differences in SI-thalamic functional connectivity between MDD and HC (MDD > HC)

Area	L/R	Cluster size, mm^3^	MNI coordinatesa			Tbvalues
			x	y	z	(peak)
central posterior gyrus	R	216	27	−33	75	3.6162^*^
central posterior gyrus	R	216	45	−30	63	3.2803^*^

*p < 0.05, single voxel threshold of *p* < 0.01 and cluster size ≥108 mm3, Alphasim correction

### Correlational analysis

**Fig. 2:**
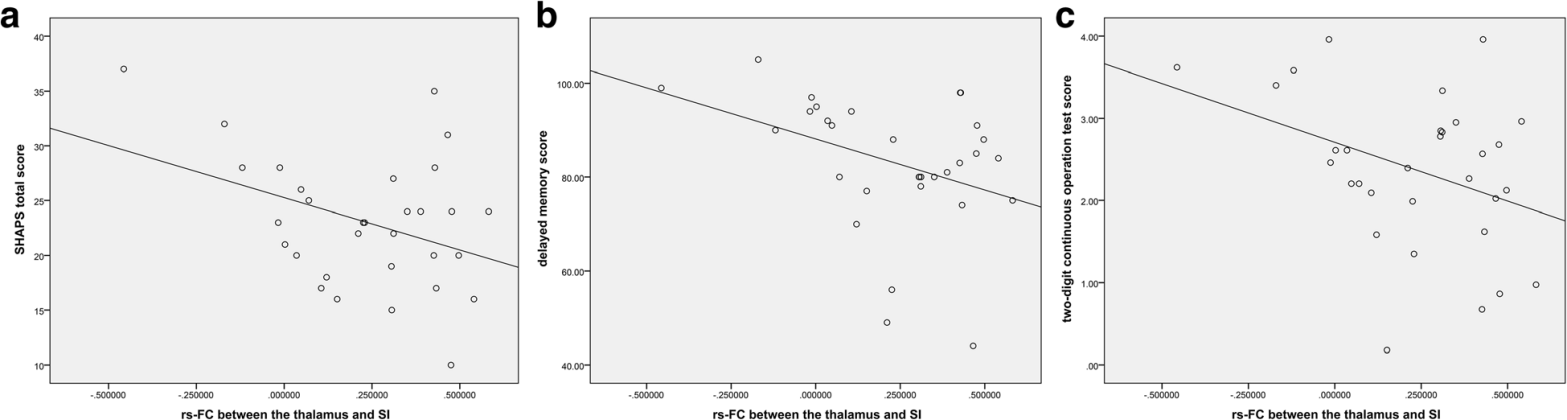
In MDD patients, brain areas significantly different levels of connectivity were negatively correlated with cognitive function, including SHAPS total score (*r* = − 0.383, *p* = 0.033), delayed memory score (*r* = − 0.376, *p* = 0.037), and two-digit continuous operation test score (*r* = − 0.369, *p* = 0.041) as shown in (**a**, **b**, and **c**), respectively

As shown in Fig. 2, significantly different brain regions and patient clinical symptoms in the SHAPS total score (*r* = − 0.383, *p* = 0.033), delayed memory score (*r* = − 0.376, *p* = 0.037), and two-digit continuous operation test score (*r* = − 0.369, *p* = 0.041) showed significant negative correlations. The significantly different brain regions had no significant correlation with the other scores.

## Discussion

Analysis of fMRI has been widely performed to study the dysfunction of brain systems in patients with psychiatric disorders. SI-thalamic functional connectivity in MDD patients has not been investigated. Our study found enhanced SI-thalamic functional connectivity, which was associated with affective experience and cognition function.

The rs-FC between the thalamus and the whole brain has been previously examined, and there were relatively consistent findings. MDD subjects had altered thalamocortical connectivity characterized by an intricate pattern in the region that was associated with clinical symptoms; indeed, one of them indicated the thalamus is hyper-connected with SI [47–50]. The importance of the SI has been gradually discovered in schizophrenia patients. For example, its abnormal internal activity predicted changes in processing speed, and external connections with the thalamus, as part of the thalamocortical-cerebellar dysconnectivity associated with schizophrenia, have become a reliable neurobiological marker [51, 52]. Our study does not emphasize the functional abnormalities of these two independent brain regions and their relationship to the development of MDD. We proposed that the differences in the SI-thalamic functional connectivity are the characteristic changes, as the central components of the experience of MDD.

Anhedonia is the most important clinical manifestation of MDD. Our study showed that abnormal connections between the thalamus and SI leads to the loss of pleasure. Numerous studies have reported that the role of SI has always been controversial with regards to the emergence of pleasure. Early studies showed that SI played an essential role in tactile pleasure perception, while studies that have used physical stimuli to modulate touch pleasantness have failed to control for differences in physical intensity and the perceived power of the overstimulation [53–55]. The research has shown that pleasant experience comes from outside of SI, but it is closely related to SI and brain activity, which may involve the thalamus [56, 57]. Our study confirmed that hyper-connection between the thalamus and SI is closely related to emotional experience, and to a certain degree, provides an explanation for the lack of pleasure experienced in MDD patients. However, further precision in identifying the role of these areas requires careful exploration of brain neuronal activity.

The starting point of our research was that cognitive function was an essential function, and our study emphasized that SI-thalamic functional connectivity is indeed closely related to attention and memory in MDD. In our research, we not only stressed the reliability of our previous theoretical basis but also provided a more detailed analysis of the cognitive items related to the SI-thalamic functional connectivity in MDD patients.

The research highlights the importance of SI for memory in different diseases or conditions [58, 59]. However, there are still some contradictory conclusions where researchers have proposed that SI did not affect the metacognitive accuracy of either temporal or spatial tactile working memory [60]. Unfortunately, the impact of thalamus and SI connections on cognition has been less explored. Research has demonstrated that in schizophrenia, hyper-connectivity with the thalamus correlated positively with working memory [61]. Our study also showed that similar changes occurred in MDD patients. The structural connection between the thalamus and SI may partially explain why the functional connections between the two are related to cognition. Thalamocortical responses provide input to SI for establishing context and storing sensory memories (e.g., slowly changing body memories stored in layer 4 and sensory memories rapidly stored in layer 2/3) [62]. However, the pathophysiological process for enhanced functional connectivity has not been elucidated.

The thalamus has a function of adjusting attention [63]. The thalamus functions as a master regulator of functional cortical connectivity. Therefore, the construction of a directed arousal state is useful for attention, impacting how a cognitive process such as attentional control unfolds over cortical space and time [64–66]. In MDD patients, we tentatively proposed that the hyper-connectivity between the thalamus and SI may disrupt the balance of this network, allowing the time for concentration to be prolonged. However, a new study shows that thalamus engagement in a delay is not to relay specific rule information but to sustain existing cortical representations [67]. These studies prompted us to carefully consider the role of thalamus inattention, and the mechanism for the inattention needs further investigation.

Our study has several limitations. First, our study design did not allow us to investigate causality or the role of change in functional connectivity between the thalamus and SI on the development of the MDD patients’ cognitive impairment. Second, the thalamus could be divided into different subregions, but we did not subdivide when selecting regions of interest. Third, the heterogeneity of our patient population and a limited number of participants may lead to biased results. Fourth, long-term follow-up observations can lead to more profound experimental results. Fifth, we did not consider the impact of the length of the initial duration of the disease on brain function connectivity.

## Conclusions

In conclusion, we have implicated enhanced SI-thalamic functional connectivity as a core feature of the pathophysiology underlying MDD. Furthermore, this abnormality is related to anhedonia, memory, and attention. The SI-thalamic functional connectivity may be a useful neural target for affective experience and cognitive interventions. Future research designed to track the effects of current treatments may develop more targeted pathophysiological therapies.

## Abbreviations

CPT: Continuous performance test
DSM-IV: Diagnostic and Statistical Manual of Mental Disorders - Fourth Edition
HAMD-17: 17-item Hamilton depression rating scale
HC: Healthy controls
MDD: Major depressive disorder
MNI: Montreal Neurological Institute
RBANS: Assessment of Neuropsychological Status
ROI: Regions of interest
rs-FC: Resting-state functional connectivity
rs-fMRI: Resting-state functional magnetic resonance imaging
SCID-I/P: The Chinese version of the Modified Structured Clinical Interview for DSM-IV TR Axis I Disorders Patient Edition
SD: Standard deviation
SHAPS: Snaith-Hamilton Pleasure Scale
SI: Primary somatosensory cortex
SPSS 23.0: IBM SPSS Statistics Version 23.0
